# Exogenous Insulin Antibody Syndrome and Subsequent Severe Subcutaneous Insulin Resistance Complicating Type 1 Diabetes

**DOI:** 10.1210/jcemcr/luaf300

**Published:** 2025-12-30

**Authors:** Vicki Cunningham, Leo Lam, Kuang-Chih Hsiao, Rosemary Ayers, Benjamin Albert, Craig Jefferies

**Affiliations:** Department of Paediatrics, Te Whatu Ora–Health New Zealand, Whangarei 0148, New Zealand; Department of Pathology and Laboratory Medicine, Te Whatu Ora–Health New Zealand, Auckland 1010, New Zealand; Pathology, Surgical and Support Services, Te Whatu Ora–Health New Zealand, Whangarei 0148, New Zealand; Department of Paediatric Immunology, Starship Child Health, Te Whatu Ora–Health New Zealand, Auckland 1050, New Zealand; Department of Paediatrics, The University of Auckland, Auckland 1050, New Zealand; Department of Paediatrics, Te Whatu Ora–Health New Zealand, Whangarei 0148, New Zealand; Liggins Institute, The University of Auckland, Auckland 1050, New Zealand; Department of Paediatric Endocrinology, Starship Child Health, Te Whatu Ora–Health New Zealand, Auckland 1050, New Zealand; Department of Paediatrics, The University of Auckland, Auckland 1050, New Zealand; Liggins Institute, The University of Auckland, Auckland 1050, New Zealand; Department of Paediatric Endocrinology, Starship Child Health, Te Whatu Ora–Health New Zealand, Auckland 1050, New Zealand

**Keywords:** diabetes, insulin, antibody, heparin, resistance, hypoglycemia

## Abstract

A young girl with type 1 diabetes mellitus (T1D) developed exogenous insulin antibody syndrome (EIAS) characterized by daytime hyperglycemia and ketosis alternating with prolonged severe nocturnal hypoglycemia. EIAS was diagnosed after the exclusion of other causes of hypoglycemia and with confirmation of very high insulin autoantibody levels, high plasma levels of bound insulin, and abnormal insulin clearance. Treatment with different insulin analogues, subcutaneous (SC) insulin pump, immunomodulation using corticosteroids, and intravenous (IV) immunoglobulin were not effective. She responded well to B-lymphocyte depletive therapy (rituximab) with a fall in insulin antibody levels and returned to usual T1D management with a marked improvement in severity of hypoglycemia. One year later she developed hyperglycemia and ketosis and showed no glycemic effect from SC insulin. She became dependent on IV insulin and was diagnosed with severe subcutaneous insulin resistance (SIR). She managed IV insulin at home but developed episodes of sepsis and central line blockage. Many treatment strategies failed, but successful management was finally achieved with the addition of heparin to insulin lispro via SC pump. EIAS and SIR are both extremely rare and, in this case, responded to very different treatment approaches.

## Introduction

Exogenous insulin antibody syndrome (EIAS) is a rare complication of type 1 diabetes mellitus (T1D), but should be considered in patients presenting with unexplained glycemic dysregulation and hyperinsulinemic hypoglycemia [1]. The diagnosis is made after the exclusion of more common causes, including surreptitious insulin administration, and is supported by the laboratory findings of high levels of insulin antibodies, high levels of bound insulin, and abnormal insulin clearance [1]. The postulated pathophysiology is insulin binding to insulin antibodies, causing insulin resistance and hyperglycemia, and later dissociation leading to hypoglycemia [2].

Severe subcutaneous insulin resistance (SIR) is characterized by severe resistance to subcutaneous (SC) insulin but normal intravenous (IV) insulin sensitivity. A range of explanations has been postulated, including abnormal absorption and insulin degradation in the SC tissue. Numerous different treatment strategies have been reported with variable outcomes and no uniform success. SIR is associated with high levels of morbidity and mortality.

There is limited published information about each of these 2 rare and severe complications of T1D. One previous case report described EIAS with concurrent features of SIR [3]. To our knowledge there are no previous reports of sequential EIAS followed by SIR.

## Case Presentation

An 8-year-old New Zealand European girl was diagnosed with T1D (positive glutamic acid decarboxylase antibodies (>2000 IU/mL) and negative anti–insulinoma-associated antigen 2 antibodies (<10 IU/mL) and was treated with insulin glargine and premeal insulin aspart, requiring 0.6 U/kg/day of insulin. She was also diagnosed with celiac disease and was managed with a gluten-free diet. Her height was in the 91st percentile, weight in the 75th percentile, and her general and skin examinations, including injection sites, were normal.

Two months post diagnosis, she developed erratic blood glucose levels following a mild gastroenteritis, with a persistent pattern of daytime hyperglycemia and frequent, prolonged nocturnal hypoglycemia.

Numerous management strategies were tried including various combinations of insulin analogues (insulin aspart, insulin lispro, insulin glargine, insulin detemir) and different methods of insulin delivery (SC injection, SC insulin pump, and deep intramuscular [IM] injection). She managed at home with once-daily insulin detemir, a low-carbohydrate diet, and intermittent corrections of hyperglycemia with IM insulin lispro. Hypoglycemia was frequent, severe, and prolonged and was managed with large amounts of oral carbohydrates, frequent SC glucagon injections, and intermittent admission for IV dextrose.

**Figure 1.**
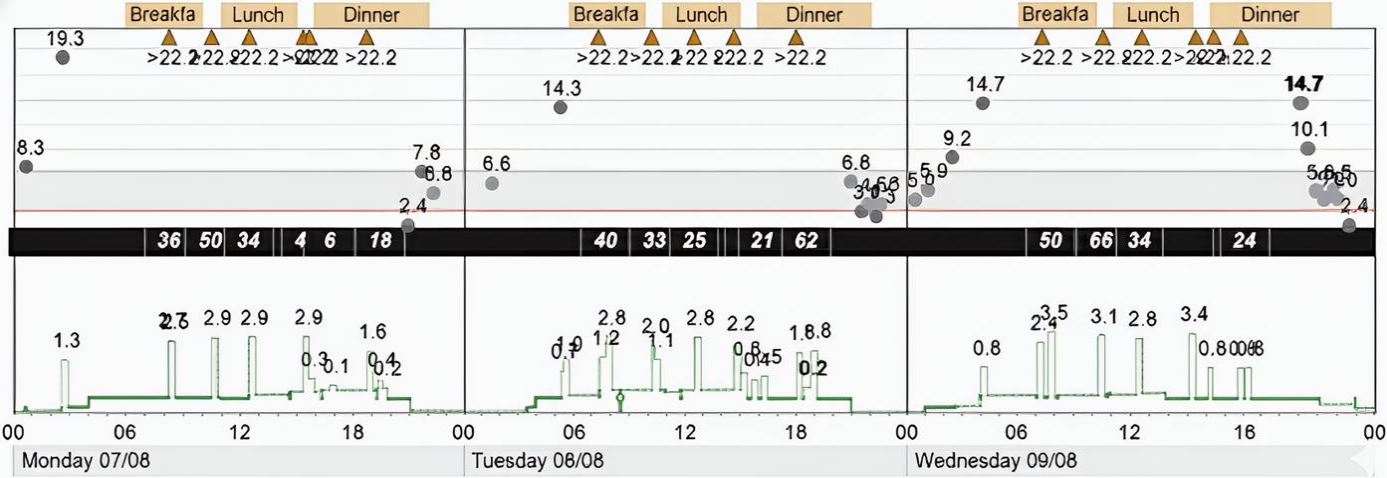
Medtronic pump data. The uploaded pump data shows blood glucose (gray circles and orange triangles) pattern of daytime hyperglycemia and nocturnal hypoglycemia. Concurrent basal and bolus insulin doses (green line) are shown, including long overnight periods when pump is suspended.

An example of the typical pattern of dysglycemia is shown in Fig. 1, when she was being treated with insulin lispro via SC insulin pump.

## Diagnostic Assessment

**Figure 2.**
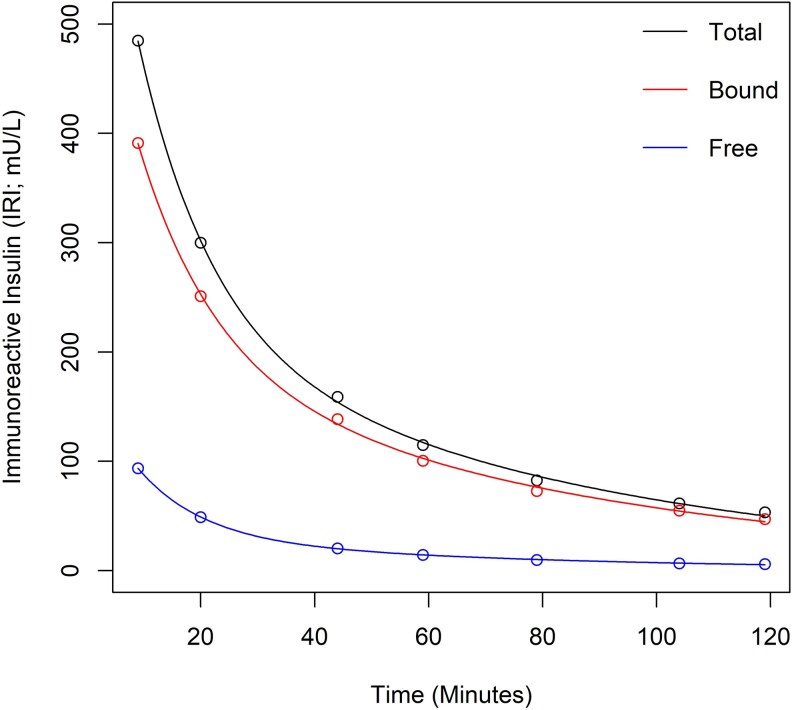
Insulin clearance study following intravenous (IV) administration of insulin (Actrapid). Insulin series was collected following intravenous administration of 3 units Actrapid over 3 minutes. The specimen was tested before (total) and after polyethylene glycol precipitation (free). The difference between the 2 results were considered as the bound insulin. The baseline insulin was 1.7 pmol/L (total) and 0.4 pmol/L (free). In the absence of insulin autoantibodies, the half-life of insulin is estimated to be in the order of minutes. All insulin measurements were performed using the Cobas, Roche method, which detects natural human insulin (Actrapid). For insulin, 6.95 mIU/L = 1 pmol/L.

At the time of hypoglycemia, the patient’s insulin level was 196 mIU/L (ADVIA Centaur; Siemens) and C peptide was undetectable (e602 Cobas, Roche). Inpatient direct observation of the patient and family ruled out surreptitious insulin administration. Cortisol, growth hormone, and metabolic responses to hypoglycemia were normal. The insulin autoantibody (IAA) level (RIA; RSR) was 330 U/mL (reference interval < 0.4 U/mL and typically <35 U/mL in treated T1D) [4]. Polyethylene glycol (PEG) recovery was low at 42% (ADVIA Centaur; Siemens), revealing high levels of bound insulin. While the patient was treated with detemir, insulin immunoreactivity was determined at 1511 mIU/L using an assay with some cross-reactivity to detemir (Iso-insulin ELISA, Mercodia). This was associated with a low PEG recovery of 2%. Insulin clearance studies were abnormal, with high levels of bound insulin persisting hours after IV insulin injection (Fig. 2; see legend for further information). Scatchard analysis of insulin autoantibodies demonstrated two populations of IAAs, with a high-affinity population demonstrating increased binding capacity (860 × 10^8^/M) and a reduced affinity (0.0015 × 10^8^/M). The autoantibody characteristics are in keeping with EIA [5].

## Treatment

Sequential immune modulatory treatment with IV immunoglobulin, IV methylprednisolone, and oral prednisolone were not effective. B-lymphocyte depletive therapy (rituximab) was given (375 mg/m^2^ body surface area/dose, weekly for 4 weeks) 2.5 years after the symptoms of EIAS began and 1 year after detection of IAAs.

## Outcome and Follow-up

**Figure 3.**
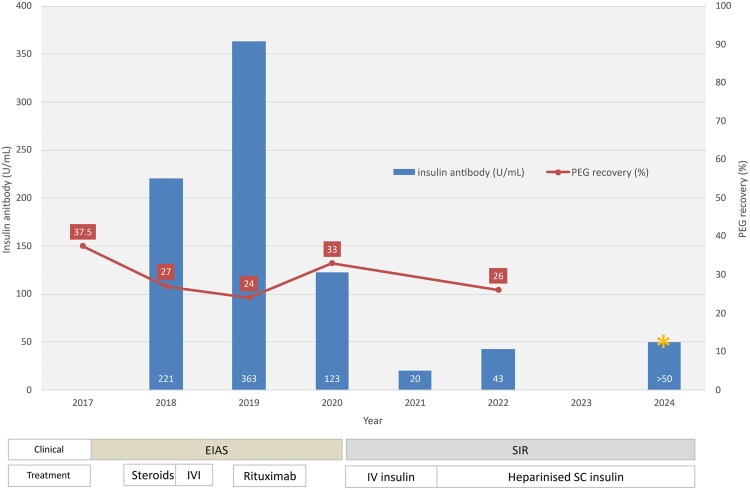
Timeline of clinical illness, treatments, insulin antibody, and polyethylene glycol (PEG) binding levels. Insulin autoantibody levels (typically <35 U/mL in treated type 1 diabetes) are shown in the blue bars. The gold asterisk denotes a level greater than 50 U/mL, which cannot be accurately graphed. This sample was tested at the same laboratory but did not undergo dilution to provide a more accurate result. PEG recovery is shown in red line graph (normal >80%). The horizontal bars at the bottom of the graph show the timeline of clinical phase and treatments used.

After rituximab, the patient’s hypoglycemia gradually improved. One year later, she resumed standard SC insulin pump therapy, with significantly fewer prolonged hypoglycemic episodes (1-2/month), glycated hemoglobin A_1c_ (HbA_1c_) of 55 mmol/mol (7.2%), and time in range (TIR, blood glucose level 4-10 mmol/L) 56% on 0.8 U/kg/day of insulin lispro. IAA levels decreased, but bound insulin remained unexpectedly high (Fig. 3).

This improvement lasted 1 year, until she was hospitalized with hyperglycemia and ketosis associated with a pump site infection. She could not be weaned off IV insulin without rapid recurrence of hyperglycemia and ketosis. High-dose SC insulin (≤5 U/kg/day) was ineffective and painful. High doses of insulin were used with caution, as concern remained that bound insulin might later dissociate and cause severe hypoglycemia; however, this did not occur. Marked SC insulin resistance was demonstrated following 20 units of SC human neutral insulin (Actrapid) with no insulin detectable in blood after 5 hours. Investigations revealed low levels of IAA but high levels of bound insulin (see Fig. 3). Her clinical and biochemical features were no longer characteristic of EIAS. This second phase was diagnosed as SIR based on severe SC insulin resistance, normal IV insulin sensitivity, and lack of measurable insulin in the bloodstream after SC injection.

Numerous SIR treatment strategies were tried, including SC insulin pump with rapid-acting insulin analogues, IM insulin, and SC dilute insulin. While IM insulin and SC insulin diluted in normal saline showed some glycemic effect on initial doses, they became ineffective after the second or third day. At times our patient had measurable insulin levels post SC administered insulin, but no glycemic effect.

This remarkable young person and her family managed IV insulin via a Hickman central line at home. They achieved target blood glucose control on 0.9 U/kg/day of IV insulin glulisine with an HbA_1c_ of 52 mmol/mol (6.9%) and TIR 79%. However, this treatment was complicated by line blockages, line infections, and episodes of sepsis. She required 3 different central lines and had increasingly difficult IV access.

After 14 months of IV insulin, and with serious concern about the ability to maintain IV access, we trialed addition of heparin to SC insulin lispro (1 mL of 5000 U/mL heparin combined with 10 mL of 100 U/mL insulin lispro). This normalized SC insulin absorption and its glycemic effect, and she was able to come off IV insulin. Our patient, now age 17 years, has been on SC heparinized insulin lispro via an insulin pump for 3 years. She remains well and is now using a hybrid closed pumping system and has an HbA_1c_ of 79 mmol/mol (9.4%) and TIR 59% on 0.6 U/kg/day of insulin. At last testing, 7 years after her initial presentation with EIAS, her IAA levels remain elevated (>50 U/mL). An overview of the timeline and treatment of our patient's complex illness is shown in Fig. 3.

## Discussion

The initial phase of our patient's illness is consistent with other reports of EIAS and showed a good response to rituximab. EIAS is characterized by unexpected hypoglycemic episodes, biochemical and clinical evidence of altered insulin pharmacokinetics, and the presence of high-affinity IAAs with altered affinity and binding characteristics. The putative mechanism leading to dysglycemia in EIAS is the presence of IAAs, which reversibly bind and dissociate with insulin, leading to unpredictable bioactive insulin in the circulation. In contrast, typical IAAs encountered with T1D irreversibly bind to insulin and therefore do not lead to dysglycemia.

Numerous immune-modulatory treatments have been used to treat EIAS [3, 6]. Our patient's EIAS was unresponsive to multiple initial immunomodulators, including corticosteroids. Further treatment with narrow-spectrum B-lymphocyte depletive therapy (rituximab) was chosen in preference to T cell–targeted agents such as mycophenolate and cyclosporin.

The pathophysiology of the subsequent SIR remains poorly understood. We considered whether systemic IAAs were the cause of the SIR phase of our patient's illness. In EIAS 2 populations of insulin antibodies have been demonstrated: the first with low affinity/high capacity (commonly associated with postprandial hyperglycemia and nocturnal hypoglycemia) and the second high affinity/low capacity (typically accompanied by severe insulin resistance) [2]. After rituximab treatment we initially postulated that the subsequent SIR phase was due to persistent high-affinity, low-capacity antibodies. We also considered tissue-specific (SC and IM) insulin antibodies leading to insulin sequestration, impaired absorption, and insulin degradation. However, other pathophysiologic explanations for SIR have also been postulated. Diabetes mellitus with resistance to insulin administered SC or IM (*DRIASM*), an alternative terminology for this condition, has been postulated to be due to tissue insulin degradation or absorption abnormalities [7].

During the SIR phase of her illness, our patient had waning response to novel insulin administration (dilute insulin and IM insulin) with repeated doses and had variable responses on insulin clearance studies. A previously reported patient with SIR had normal response to tissue resistance test with a single dose but showed variable results with a prolonged tissue resistance test with repeated dosing over 3 days, and the authors postulated a gradual buildup to SC resistance [3].

Numerous different treatment strategies have been used for SIR, with variable success in different reports. In some cases SIR has been effectively managed with alternative methods of insulin delivery, for example, inhaled insulin and peritoneal insulin, but these were not available to us in New Zealand [8-10]. Successful pancreatic transplant has been undertaken in several reported cases [7, 10]. Tokuyama et al and Nakamura et al [11, 12] each report a young woman with T1D and SIR successfully treated with heparinized insulin lispro via continuous SC insulin infusion. However, unlike our patient, the patient described by Tokuyama et al [11] was negative for antibodies to human insulin. The patient described by Nakamura et al [12] did not have these investigations reported. However, the combination of insulin lispro with heparin was ineffective in other case reports and similarly coadministration with protease inhibitors, such as topical nafamostat ointment or aprotinin, were effective in some, but not all case reports [8-10, 13, 14]. Recently, ultrarapid insulin lispro containing treprostinil and citrate has also been used in SIR with success [15]. The low levels of circulating insulin antibodies during the SIR phase of our patient's illness led us to trial heparinized insulin in favor of further immunomodulatory therapy. The subsequent successful management with heparinized SC insulin supports the concept of tissue-specific factors leading to insulin degradation or absorption abnormalities in our case, with heparin acting as a chaperone through the SC tissues.

Our case presentation adds to the growing body of information about the recognition and treatment of EIAS. However, the etiology of SIR and its management remain poorly understood. It is unclear what role systemic IAAs had in the SIR phase of our patient's illness or if this phase was due to unrelated tissue-specific factors in skin and muscle leading to impaired absorption, sequestration, and degradation of insulin. Our patient has had a good outcome and currently remains well.

## Learning Points

- EIAS is a rare complication of T1D characterized by inexplicable erratic blood glucose levels and severe hyperinsulinemic hypoglycemia.
- Rituximab effectively treated EIAS, with its full effect seen after 12 months.
- In our case SIR developed following successful treatment of EIAS.
- The etiology of SIR remains poorly understood.
- There are no guidelines for the management of SIR, though in our case SC heparinized insulin was effective and obviated the need for more-invasive treatments.

## Data Availability

Some or all datasets generated during and/or analyzed during the current study are not publicly available but are available from the corresponding author on reasonable request.

## Abbreviations

EIAS: exogenous insulin antibody syndrome
HbA_1c_: glycated hemoglobin A_1c_
IAA: insulin autoantibody
IM: intramuscular
IV: intravenous
PEG: polyethylene glycol
SC: subcutaneous
SIR: subcutaneous insulin resistance
T1D: type 1 diabetes
TIR: time in range
